# Association of clinical features and myositis-specific antibodies in idiopathic inflammatory myopathy: a retrospective study from southern China

**DOI:** 10.3389/fimmu.2025.1674437

**Published:** 2025-11-06

**Authors:** Can Li, Yushi Zheng, Yu Zhang, Yujin Ye, Hui Zhang, Niansheng Yang, Shuang Wang

**Affiliations:** 1 Department of Rheumatology, First Affiliated Hospital of Sun Yat-sen University, Guangzhou, China; 2 Department of Medicine, Traditional Chinese Medicine Hospital of Laifeng County, Enshi, Hubei, China

**Keywords:** idiopathic inflammatory myopathy, myositis-associated autoantibody, myositis-specific autoantibody, interstitial lung disease, malignancy, cardiac involvement, hyperlipidemia

## Abstract

This study aimed to investigate the profiles of myositis-specific autoantibodies (MSA) and their correlation with distinct clinical features in patients with idiopathic inflammatory myopathy (IIM) in southern China. We retrospectively analyzed the medical records of 208 IIM patients, collecting data on their demographic variables, clinical manifestations, comorbidities, and MSA test results. Of the 208 patients, 185 were positive for MSAs. 69 patients were anti-MDA5 positive, 61 patients were anti-ARS positive followed by anti-SRP (34), anti-TIF1-γ (26), anti-Mi-2β (10), anti-NXP2 (10), anti-HMGCR (9), anti-Mi-2α (6), anti-cN-1A (6), and anti-SAE1 (1). Distinct clinical phenotypes were strongly associated with specific antibodies. Anti-MDA5 positive patients had shorter disease duration, less muscle involvement, but higher rates of rash, alopecia, arthritis, fever, and ILD with poorer prognosis. Anti-ARS positive patients had longer disease duration, mechanic’s hands, arthritis, fever, and ILD, but better prognosis. Both anti-MDA5 and anti-ARS antibodies were independent risk factors for developing ILD. Anti-TIF1-γ and anti-Mi-2 were most detected in IIM patients combined with malignancies, and nasopharyngeal carcinoma was the most common malignant tumor. Furthermore, hyperlipidemia and elevated cardiac biomarkers were frequently observed, particularly in patients positive for anti-SRP. The 3-month survival rate for anti-MDA5 positive patients was 87.8%, with all deaths attributed to rapidly progressive-ILD (RP-ILD). In contrast, other antibody positive patients had a 100% survival rate. This comprehensive analysis of a southern Chinese IIM cohort underscores that MSA profiles can effectively stratify patients into clinically distinct subgroups, which is crucial for predicting specific organ involvement, prognosis, and developing tailored treatment strategies.

## 1 Introduction

IIM is a heterogeneous group of autoimmune diseases characterized by muscle weakness and a range of extramuscular manifestations including skin, lung, joint, heart, and other organ involvement. Some patients suffer from severe visceral involvement and comorbidities such as interstitial lung disease (ILD), cardiac involvement and associated malignancy which are major causes affecting the quality of life and mortality (1).

Approximately 60% of IIM patients have autoantibodies targeting their own tissues and cells, which are categorized into MSA and myositis-associated autoantibodies (MAA) (2). MSAs include anti-aminoacyl tRNA synthetases (ARS) (anti-Jo-1, anti-PL-7, anti-PL-12, anti-EJ, anti-OJ), anti-MDA5, anti-TIF1-γ, anti-SAE1, anti-NXP2, anti-cN-1A, anti-HMGCR, anti-Mi-2α, anti-Mi-2β antibodies, while MAAs include anti-Ku, anti-Ro52, anti-PM-Scl100, anti-PM-Scl75 antibodies, etc. (3). Depending on clinical and laboratory findings, especially the presence of MSA, muscle biopsy may not be necessary in every patient suspected of an IIM for diagnosis. MSA are also associated with particular clinical manifestations within the IIM spectrum. Anti-MDA5 is strongly associated with ILD, including a rapidly progressive phenotype (4, 5). Anti-ARS antibodies are strongly associated with interstitial lung disease, Raynaud ‘s phenomenon, arthritis, and mechanic’s hands (6).

Given the uneven distribution of MSA and the diversity of clinical manifestations in the IIM spectrum, this study aimed to investigate the MSA profiles and its correlation with clinical features in IIM patients in southern China.

## 2 Materials and methods

### 2.1 Patient

We conducted this retrospective cohort study in the department of rheumatology and immunology at the First Affiliated Hospital of Sun Yat-sen University. The study included 208 IIM patients aged 18 to 75 years and hospitalized between November 2018 and October 2023 and collected information from their first hospitalization. 57.2% of these IIM patients met 2017 EULAR/ACR ‘definite’ or ‘probable’ classification criteria (7). Others who did not meet this criterion either meet the Bohan and Peter ‘definite’ or ‘probable’ diagnostic criteria (8, 9), or met the antisynthetase syndrome criteria (10). Exclusion criteria targeted patients who did not meet the classification criteria, those with severe infections, and those with other connective tissue diseases (such as systemic lupus erythematosus, Sjogren’s syndrome, systemic sclerosis, rheumatoid arthritis). The study has been granted ethical approval by the First Affiliated Hospital of Sun Yat-sen University and has followed the principles of the Declaration of Helsinki throughout the research process.

### 2.2 Data collection

The study collected data on demographic characteristics, clinical presentations, and laboratory tests. IIM-related ILD was diagnosed using high HRCT with abnormalities consistent with ILD, i.e., parenchymal micronodules and nodules, linear opacities, irregularity of the interfaces between peripheral pleura and aerated lung parenchyma, ground-glass opacities, honeycombing, and traction bronchiectases or bronchiolectases (11). Diagnosis was confirmed by two radiologists and one immunologist. RP-ILD was defined to meet criteria including imaging manifestations and lung symptoms had worsened within 3 months or the lung function had markedly worsened since the previous test (e.g., the FVC decreased by >10% and the partial arterial oxygen pressure decreased by >10 mmHg) (12). The diagnostic criteria for hyperlipidemia followed the clinical classification of the 2023 edition of the “Chinese Guidelines for the Prevention and Treatment of Dyslipidemia in Adults” (13).

### 2.3 Autoantibody detection

Sera from IIM patients were sent to an external laboratory (EUROIMMUN Medical Laboratory, Guangzhou, China) for MAA and MSA testing. 14 MSAs: anti-MDA5, anti-Mi-2α, anti-Mi-2β, anti-TIF1-γ, anti-NXP2, anti-SAE1, anti-SRP, anti-HMGCR, anti-cN-1A, anti-ARS (anti-Jo-1, anti-PL-7, anti-PL-12, anti-EJ, anti-OJ) and 4 MAAs: anti-Ku, anti-Ro52, anti-PM-Scl75, anti-PM-Scl100 were analyzed using an immunoblotting kit (catalog number: DL_1530-8_G_LS, EUROIMMUN, Hangzhou, China). Positive (+), moderately positive (++), and strongly positive (+++) were considered positive results for autoantibodies.

### 2.4 Grouping design

Based on the myositis antibody profile test, the included IIM patients were categorized into three groups: those with single anti-MDA5 antibody positivity (Group I), those with single anti-ARS antibody positivity (Group II), and those with other MSAs excluding anti-MDA5 and anti-ARS (Group III). Furthermore, patients were stratified into two subgroups: IIM-ILD and IIM-non ILD groups based on the presence or absence of ILD.

### 2.5 Statistical methods

Data were analyzed using SPSS 26.0 statistical software and the R programming language. Normally distributed continuous variables were expressed as the mean ± standard deviation, and group comparisons were made using the t-test. Skewed continuous variables were expressed using the median (M) with the 25th and 75th percentiles (P25, P75), and group comparisons were made using the Mann-Whitney U rank-sum test. Categorical data were expressed as number of cases (n) and percentage (%), and group comparisons were made using the chi-square test or Fisher’s exact test. For multiple samples with non-normally distributed continuous variables, the Kruskal-Wallis H test was used, and for normally distributed variables, analysis of variance (ANOVA) was used. Pairwise comparisons between subgroups were made using the Bonferroni correction test. Logistic regression analysis was used to analyze the risk factors associated with IIM-ILD, and Kaplan-Meier survival analysis was used to assess the impact of autoantibodies on prognosis. P-value less than 0.05 indicated a statistically significant difference.

## 3 Results

### 3.1 Myositis-specific antibody distribution

A total of 208 patients diagnosed with IIM were included in this study. The cohort comprised 145 females (69.7%) and 63 males (30.3%), with a median disease duration of 6.00 (3.00, 24.0) months. The median age at diagnosis was 50.0 (41.0, 57.2) years (**Supplementary Table S1**).

**Figure 1:**
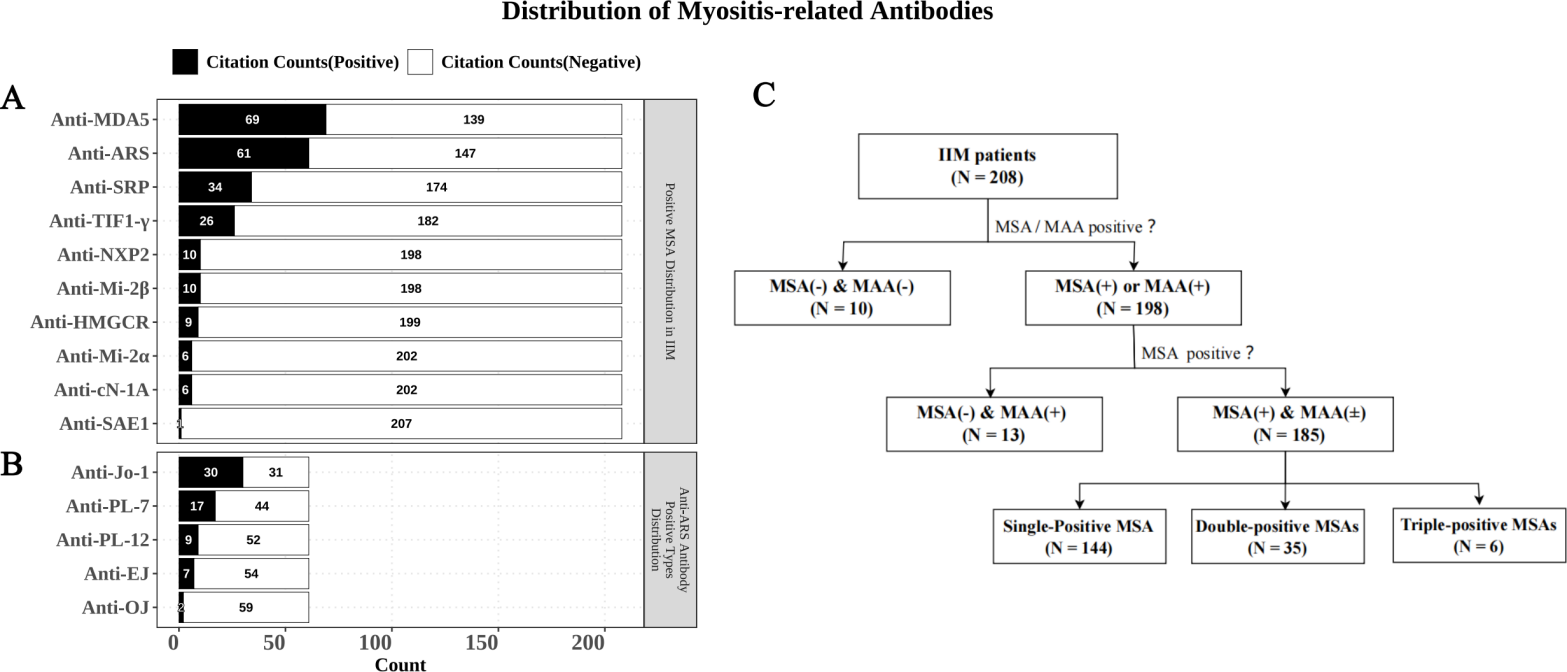
The distribution of myositis-related antibodies in IIM patients. **(A)** The distribution of MSAs in 208 IIM patients. **(B)** Distribution of various anti-ARS antibodies in anti-ARS positive patients. **(C)** Profiles of MSA and MAA positivity in IIM patients.

Among the 208 patients with complete myositis antibody test results, 185 (88.9%) were positive for at least one MSA. The distribution of MSA positivity in the cohort is illustrated in **Figure 1A**. The most frequently detected antibody was anti-MDA5 (n = 69), followed by anti-ARS (n = 61). Distribution of various anti-ARS antibodies in anti-ARS positive patients is shown in **Figure 1B**, with the highest positive rate observed for anti-Jo-1 (n = 30), followed by anti-PL-7 (n = 17), anti-PL-12 (n = 9), anti-EJ (n = 7), and anti-OJ (n = 2). The positivity rates of other MSAs, in descending order, were anti-SRP (n = 34), anti-TIF1-γ (n = 26), anti-NXP2 (n = 10), anti-Mi-2β (n = 10), anti-HMGCR (n = 9), anti-Mi-2α (n = 6), anti-cN-1A (n = 6), and anti-SAE1 (n = 1) (**Figure 1A**). Notably, 144 patients exhibited isolated MSA positivity, while 35 and 6 patients demonstrated dual and triple MSA positivity, respectively. 13 patients tested negative for MSA but positive for MAA, and 10 patients were negative for both MSA and MAA (**Figure 1C**). The antibody profiles and clinical characteristics of multi-positive patients have been summarized in **Supplementary Figure S1** and **Supplementary Table S2**.

### 3.2 Demographic data and clinical manifestations of different IIM groups

Patients were categorized into three groups based on MSA profiles: Group I (isolated anti-MDA5-positive, n = 50), Group II (isolated anti-ARS-positive, n = 38), and Group III (other MSA-positive, n = 61). Demographic comparisons revealed no significant differences in age (*P* = 0.752) or gender distribution (*P* = 0.705) among the three groups. However, the disease duration in group II was the longest, with an average of 18 months (3–26 months), while that in group I was the shortest, with an average of 5 months (2–10 months) (**Table 1**).

**Table 1:** Demographic data and clinical manifestations in three IIM subgroups.

Clinical feature	Group I (N = 50)	Group II (N = 38)	Group III (N = 61)	P-value
Age (years)	49 (40, 55)	50 (41, 58)	51 (42, 57)	0.752
Gender (Female)	37 (74%)	25 (66%)	43 (70%)	0.705
Disease Duration (months)	5 (2, 10)	18 (3, 26)	10 (3, 28)	0.002
	*P < 0.001	#P = 0.278	**P = 0.008	
Muscle Weakness (%)	25 (50%)	20 (53%)	50 (82%)	<0.001
	*P = 0.977	#P = 0.006	**P = 0.002	
Myalgia (%)	17 (34%)	15 (39%)	30 (49%)	0.259
Dysphagia for Liquids (%)	3 (6.0%)	2 (5.3%)	7 (11%)	0.588
Swallowing Difficulty (%)	4 (8.0%)	3 (7.9%)	17 (28%)	0.005
	*P > 0.999	#P = 0.016	**P = 0.008	
Heliotrope Rash (%)	38 (76%)	7 (18%)	27 (44%)	<0.001
	*P < 0.001	#P = 0.016	**P = 0.002	
Gottron’s Sign/Papules (%)	24 (48%)	4 (11%)	7 (11%)	<0.001
	*P < 0.001	#P > 0.999	**P < 0.001	
Shawl Sign (%)	12 (24%)	0 (0%)	10 (16%)	0.006
	*P = 0.01	#P = 0.018	**P = 0.447	
Mechanic’s Hands (%)	5 (10%)	6 (16%)	1 (1.6%)	0.025
	*P = 0.52	#P = 0.036	**P = 0.133	
Raynaud’s Phenomenon (%)	2 (4.0%)	3 (7.9%)	0 (0%)	0.063
Skin Ulcers (%)	9 (18%)	1 (2.6%)	0 (0%)	<0.001
	*P = 0.058	#P = 0.999	**P = 0.015	
Arthritis (%)	24 (48%)	17 (45%)	5 (8.2%)	<0.001
	*P = 0.93	#P < 0.001	**P < 0.001	
Alopecia (%)	9 (18%)	3 (7.9%)	1 (1.6%)	0.008
	*P = 0.292	#P = 0.234	**P = 0.015	
Fever (%)	16 (32%)	10 (26%)	0 (0%)	<0.001
	*P < 0.732	#P < 0.001	**P < 0.001	

*A comparison between groups I and II, #A comparison between groups II and III, **A pairwise comparison between groups I and III. *P* < 0.05 indicates a statistically significant difference.

Significant intergroup differences were observed for muscle weakness, swallowing difficulty, cutaneous manifestations including heliotrope rash, Gottron’s sign, shawl sign, mechanic’s hands, and skin ulcers, as well as arthritis, alopecia and fever. However, no statistically significant differences were found among the groups regarding myalgia, dysphagia for liquids, or Raynaud’s phenomenon. Group III had the highest prevalence of muscle weakness and swallowing difficulty. Arthritis and fever were more prevalent in Groups I and II compared to Group III, whereas no significant difference was observed between Groups I and II. The prevalence of heliotrope rash, Gottron’s sign, and shawl sign was highest in Group I. Mechanic’s hands were most frequent in Group II. Additionally, Group I exhibited a significantly higher frequency of skin ulcers and alopecia.

Baseline laboratory tests for muscle enzymes and inflammation markers were shown in **Table 2**. Generally, CK and LDH of group III patients were significantly higher than those of group I and group II. In contrast, the chronic inflammatory markers of ferritin, ESR, and serum amyloid A (SAA) in Groups I and II were higher than those in Group III, and the elevation was more significant in Group I. However, there was no significant difference in CRP levels among the three groups, partly because these IIM patients had already started anti-inflammatory treatments such as glucocorticoids, and the CRP with the shortest half-life was already in a declining stage.

**Table 2:** Baseline laboratory tests in three IIM subgroups.

Laboratory investigations	Group I (N = 50)	Group II (N = 38)	Group III (N = 61)	P-value
AST (U/L)	64.0 [40.2, 100]	33.0 [22.2, 69.2]	77.0 [44.0, 164]	0.001
	*P = 0.003	#P < 0.001	**P = 0.245	
ALT (U/L)	54.0 [31.8, 85.0]	30.5 [16.2, 67.8]	66.0 [30.0, 129]	0.004
	*P = 0.016	#P = 0.002	**P = 0.177	
LDH (U/L)	316 [280, 451]	296 [237, 512]	484 [338, 856]	<0.001
	*P = 0.548	#P = 0.002	**P < 0.001	
CK (U/L)	62.0 [35.2, 131]	241 [72.0, 1473]	1074 [262, 3135]	<0.001
	*P < 0.001	#P = 0.021	**P < 0.001	
Ferritin (ug/L)	730 [308, 1339]	189 [103, 507]	153 [74.6, 303]	<0.001
	*P = 0.003	#P = 0.330	**P < 0.001	
CRP (mg/L)	3.27 [0.82, 10.6]	2.80 [0.80, 24.4]	2.05 [0.84, 4.75]	0.491
	*P = 0.513	#P = 0.273	**P = 0.485	
ESR (mm/h)	35.0 [23.0, 53.0]	29.5 [12.5, 51.0]	25 [10.0, 38.0]	0.032
	*P = 0.282	#P = 0.294	**P = 0.007	
SAA (mg/L)	24.7 [12.10, 87.2]	17.4 [7.19, 79.3]	9.51 [4.23, 26.1]	0.047
	*P = 0.632	#P = 0.117	**P = 0.015	

*A comparison between groups I and II, #A comparison between groups II and III, **A pairwise comparison between groups I and III. P < 0.05 indicates a statistically significant difference.

### 3.3 Comorbidities in different IIM groups

**Figure 2:**
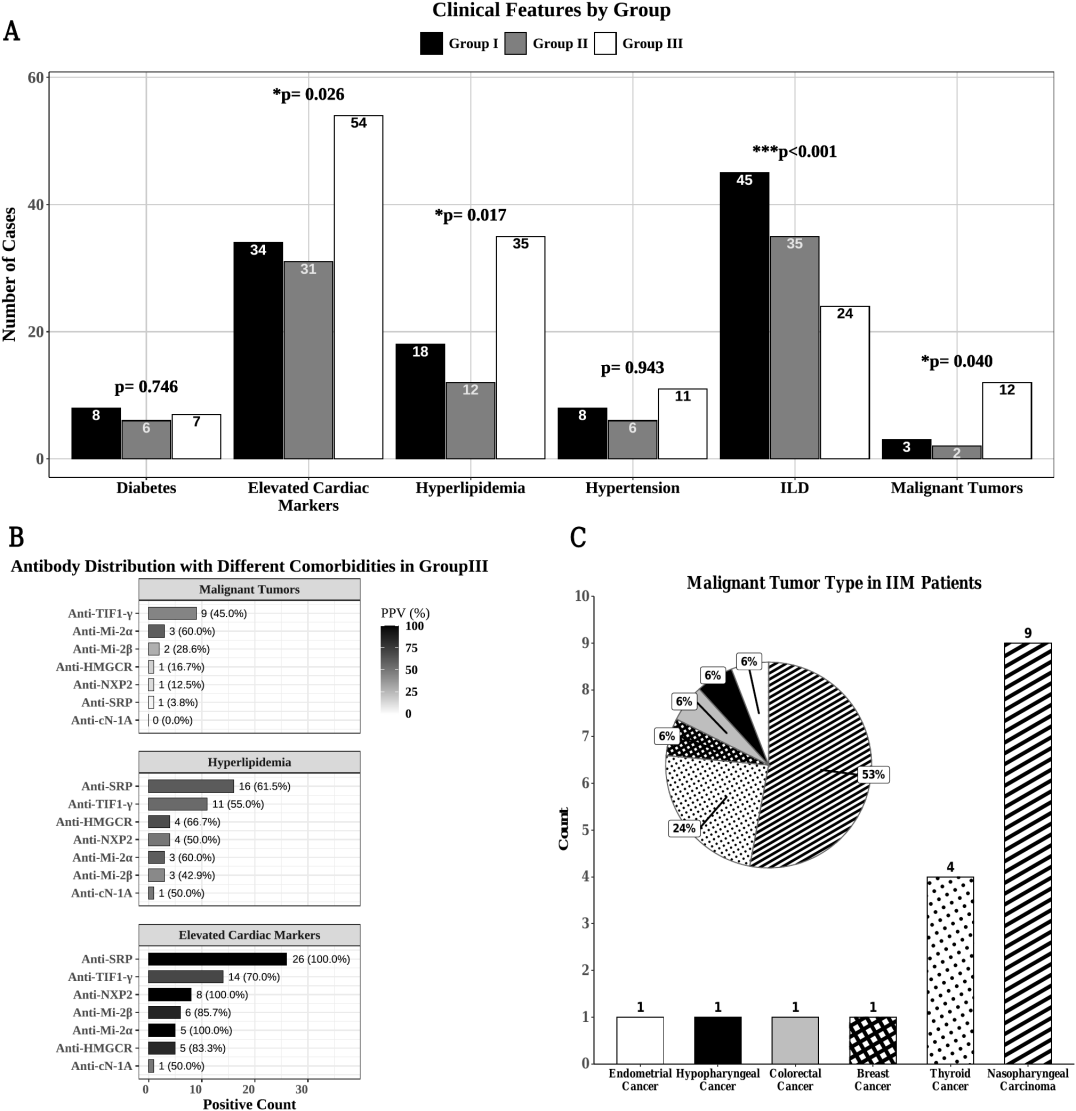
Clinical comorbidities in IIM patients. **(A)** Clinical comorbidities of IIM patients in three MSA subgroups. **(B)** Distribution of MSA in group III patients combined with malignancies, hyperlipidemia, and elevated myocardial markers. **(C)** Distribution of malignant tumor types in IIM patients. *p<0.05, ***p<0.001.

There were significant differences among the three groups in the incidence of malignancy, hyperlipidemia and elevated myocardial markers, but no significant differences in the incidence of hypertension and diabetes (**Figure 2A**).

Group III exhibited the highest incidence of malignancy at 19.7% (12/61). Notably, anti-TIF1-γ was the most frequently detected antibody in patients with malignancy (n = 9), demonstrating a positive predictive value (PPV) of 45.0% (Proportion of patients with malignancy among anti-TIF1-γ-positive patients). The distribution of other MSA types in malignancy cases included anti-Mi-2α (n = 3, 60.0%), anti-Mi-2β (n = 2, 28.6%), anti-HMGCR (n = 1, 16.7%), anti-NXP2 (n = 1, 12.5%), and anti-SRP (n = 1, 3.8%) (**Figure 2B**). The distribution of malignancies is presented in **Figure 2C**, with nasopharyngeal cancer (NPC) being the most common (n = 9), followed by thyroid cancer (n = 4), breast cancer (n = 1), colon cancer (n = 1), hypopharyngeal cancer (n = 1), and endometrial cancer (n = 1). The temporal relationship with IIM onset, screening modalities, and follow-up outcomes for each malignancy were summarized in **Supplementary Table S3**. 88.2% (15/17) of the malignancies were diagnosed within three years before or after the onset of myositis. 64.7% (11/17) of the malignancies were diagnosed at the same time as the onset of myositis. Among the 9 patients with NPC, 4 were diagnosed after nasal endoscopy screening and 5 were diagnosed after whole-body PET-CT screening. All thyroid cancers were diagnosed after thyroid ultrasound screening.

As shown in **Figure 2A**, a total of 43.6% (65/149) of IIM patients in the three subgroups had hyperlipidemia. 16.9% (11/65) of patients with hyperlipidemia have their lipid captured before glucocorticoids use, while 83.1% (54/65) of patients have their blood lipid levels measured after glucocorticoids use without use of other dyslipidemia medications. Except for two patients who used statins for secondary prevention of coronary heart disease, none of these hyperlipidemic patients had statin exposure before the onset of myositis, including those positive for anti-HMGCR. Hyperlipidemia was notably prevalent in Group III (57.4%, 35/61). The antibody distribution in hyperlipidemic patients within Group III showed that anti-SRP antibodies were the most frequently detected (n = 16), with 61.5% PPV, followed by anti-TIF1-γ (n = 11, 55.0%), anti-HMGCR (n = 4, 66.7%), anti-NXP2 (n = 4, 50.0%), anti-Mi-2α (n = 3, 60.0%), anti-Mi-2β (n = 3, 42.9%), and anti-cN-1A (n = 1, 50.0%) (**Figure 2B**).

Elevation of cardiac markers including creatine kinase-MB (CK-MB), myoglobin (MYO), troponin T (TnT), N-terminal pro-B type natriuretic peptide (NT-proBNP), indicative of myocardial involvement, was significantly more frequent in Group III (88.5%, 54/61) than in the other two groups (*P* = 0.026). The most commonly associated autoantibody in these patients was anti-SRP (n = 26), with PPV of 100.0%, followed by anti-TIF1-γ (n = 14, 70.0%), anti-NXP2 (n = 8, 100.0%), anti-Mi-2β (n = 6, 85.7%), anti-Mi-2α (n = 5, 100.0%), anti-HMGCR (n = 5, 83.3%), and anti-cN-1A (n = 1, 50.0%) (**Figure 2B**).

### 3.4 MSA distribution in IIM-related ILD

**Figure 3:**
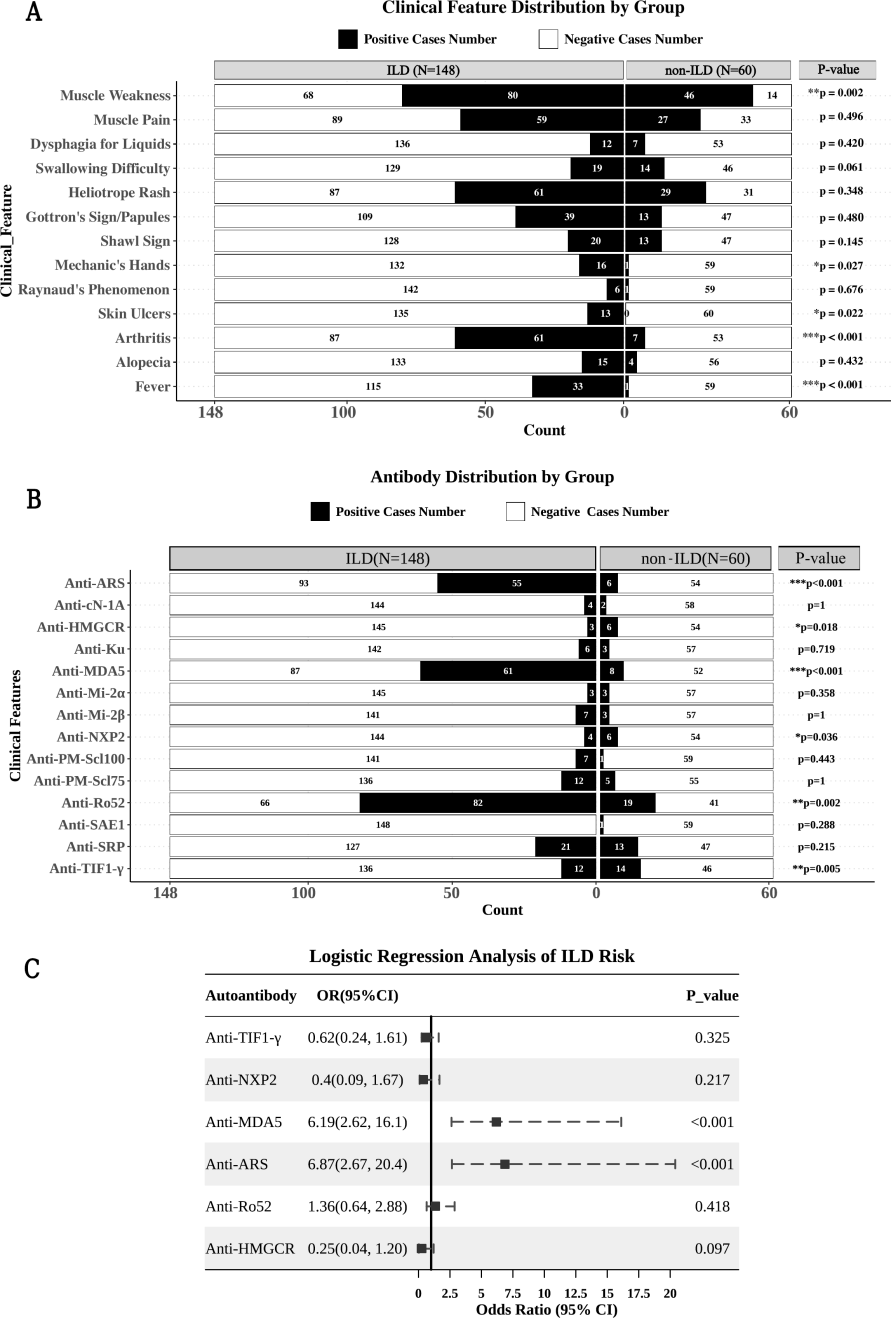
Clinical features, autoantibodies distribution and risk antibodies of IIM patients with ILD. **(A)** The clinical features of IIM patients with and without ILD. **(B)** Distribution of MSA and MAA in IIM patients with and without ILD. **(C)** Logistic regression analysis of the risk of ILD in patients with different myositis antibodies. *p<0.05, **p<0.01, ***p<0.001.

ILD was highly prevalent in Groups I and II, with incidence rates of 90.0% (45/50) and 92.1% (35/38), respectively, which were significantly higher than that in Group III (39.3%, 24/61) (*P* < 0.001) (**Figure 2A**). Baseline FVC/DLCO results in ILD patients of three groups were presented in **Supplementary Table S4**. FVC%pred in ILD patients of the three groups were 74.4 ± 13.0, 68.7 ± 18.5 and 76.7 ± 17.1 (*P* = 0.297). DLCO% of Group I and Group II were 52.0 [49.2, 71.0] and 58.5 [52.0, 66.5] respectively, which were significantly lower than that of Group III (79 [74.5, 86.5]) (*P* = 0.013). Analysis of the clinical manifestations of all IIM (n = 208) patients with and without ILD (n = 60) revealed that IIM patients with ILD (n = 148) more often experienced fever, arthritis, skin ulcers and mechanic’s hands but less often experienced muscle weakness (**Figure 3A**).

Autoantibody analysis revealed that the ILD group had significantly higher positivity rates for anti-MDA5, anti-ARS, and anti-Ro52 antibodies (*P* < 0.05). In contrast, non-ILD patients exhibited significantly higher positivity rates for anti-TIF1-γ, anti-NXP2, anti-HMGCR antibodies (*P* < 0.05). The positivity rates of anti-Mi-2α, anti-Mi-2β, anti-SRP, anti-SAE1, anti-Ku, anti-cN-1A, anti-PM-Scl100, and anti-PM-Scl75 antibodies did not differ significantly between the two groups (**Figure 3B**).

Multivariate logistic regression analysis (**Figure 3C**) identified only anti-MDA5 (OR = 6.19, *P* < 0.001) and anti-ARS (OR = 6.78, *P* < 0.001) as independent risk factors for ILD in IIM patients. Conversely, positivity for anti-TIF1-γ (OR = 0.62, *P* = 0.325), anti-NXP2 (OR = 0.4, *P* = 0.217) and anti-HMGCR (OR = 0.25, *P* = 0.097) was negatively correlated with ILD occurrence. However, these associations were not statistically significant.

### 3.5 Survival analysis of IIM patients

**Figure 4:**
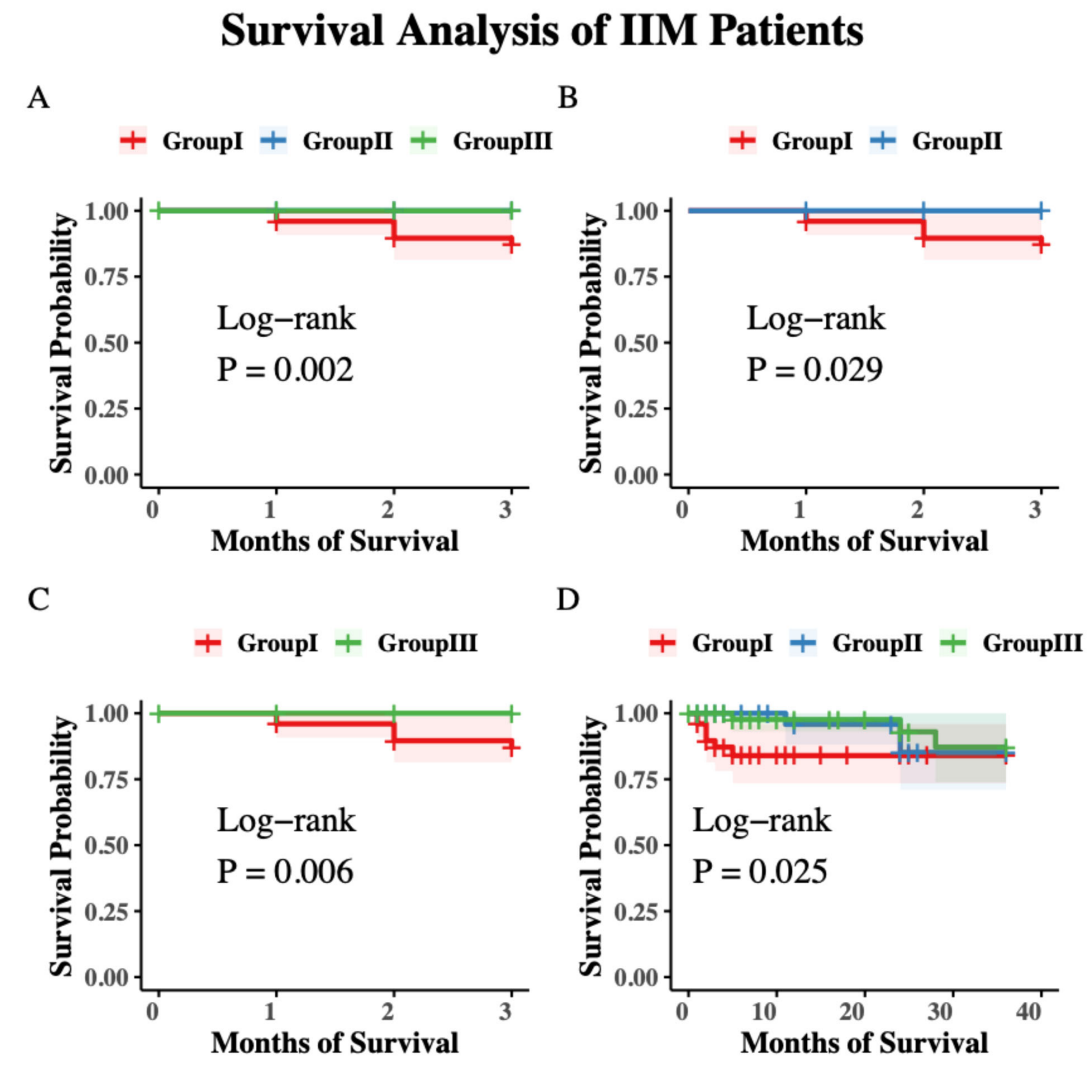
Survival analysis of IIM patients in three MSA subgroups. **(A)** 3-month(short-term) survival rates and comparison between Groups I, II, and III patients. **(B)** Short-term survival rates and comparison between Groups I and II patients. **(C)** Short-term survival rates and comparison between Group I and III. **(D)** 36-month (long-term) survival rates and comparison between Groups I, II, and III patients.

The median follow-up duration was 23 months (range: 0-64 months). Kaplan-Meier survival analysis at 3 months revealed significant differences in survival probabilities among Groups I, II, and III (*P* = 0.002) (**Figure 4A**). The 3-month survival rate for Group I was 87.8%, with all deaths attributed to RP-ILD. In contrast, the survival rates for Groups II and III were both 100%. Statistically significant differences were observed between Group I and both Group II (*P* = 0.029) and Group III (*P* = 0.006) (**Figures 4B, C**). In the extended survival analysis at 36-month, overall survival differences among the groups remained significant (*P* = 0.025) (**Figure 4D**). The treatment regimens for these three groups with ILD and without ILD were summarized in **Supplementary Table S5**.

## 4 Discussion

In this retrospective study of a southern Chinese cohort, we demonstrate that MSAs are powerful tools for stratifying patients with IIM into distinct clinical and prognostic subgroups. Our primary findings reveal a unique MSA distribution dominated by anti-MDA5 and anti-ARS antibodies. These two antibodies are independent risk factors for ILD, yet they are associated with markedly different clinical courses and survival outcomes. Furthermore, we found that other MSAs are closely associated with specific comorbidities, including a significant link between anti-TIF1-γ and NPC, highlighting a regional malignancy pattern, as well as a high incidence of cardiac involvement and hyperlipidemia in patients positive for other MSAs (Group III).

MSAs have been detected in the sera of 50-70% IIM patients and are of great value in the diagnosis, classification, treatment guidance and prognosis of the disease (2, 14). With the increasing types of detectable MSAs, the positivity of MSA in IIM patients reaches 80-90%. However, there are significant differences in the distribution of antibody spectra among different populations. A striking feature of our cohort is the high prevalence of anti-MDA5 antibodies (33.2%), making it the most common MSA observed, followed by anti-ARS (29.3%). This contrasts sharply with a report on myositis in a Caucasian population, where anti-MDA5 positivity is rare (1.1%) (15), and anti-cN-1A was most common in 19.8% adult-onset IIM, followed by anti-Jo-1(16.8%). Another study from Spain reported 12% prevalence of anti-MDA5 in 117 DM patients (16). In a study from Japan, of 84 IIM patients, 31 (36.9%) were positive for anti-ARS, 18 (21.4%) patients were positive for anti-MDA5 (17). In China, studies from different regions also showed the diversity of MSA distribution. In a study from north of China, anti-ARS with a frequency of 18.7% was the most frequently detected followed by Anti-TIF1-γ (14.3%) and anti-MDA5 (12.5%). In our cohort, most frequently detected antibody was anti-MDA5 (33.2%), followed by anti-ARS (29.3%). MSAs were thought to be mutually exclusive in IIM patients. However, detection of multiple MSAs is increasingly reported (18–20). In our study, 19.7% (41/208) IIM patients were positive for at least 2 MSAs, including 6 patients who were triple positive for MSAs.

Subgroup analysis based on MSA typing showed remarkably higher incidence of fever, arthritis, and ILD in the anti-MDA5 and anti-ARS groups than that in the other antibody-positive group (group III). The anti-MDA5 group always manifested with typical cutaneous findings (Gottron’s sign, heliotrope eruption and shawl sign) and characteristic ulcerative rash. These obvious skin manifestations plus some RP-ILD are the main reasons for the shortest disease duration among the three groups. Compared with group III, anti-ARS group had no significant muscle weakness and elevated muscle enzymes, and when compared with MDA5 group, there was no typical rash. Although mechanic’s hands exhibit certain specificity, they are often overlooked. These factors led to delayed diagnosis in the anti-ARS group, making its disease course the longest among the three groups. Occasionally, antisynthetase syndrome, which presents primarily with polyarthritis, can be misdiagnosed as seronegative rheumatoid arthritis (21).

A well-established clinical concern is the association between IIM and malignancy, particularly in dermatomyositis (DM) and, to a lesser extent, polymyositis (PM). Certain MSAs exhibit strong correlations with cancer-associated myositis (CAM). Anti-TIF1-γ is most strongly linked with CAM with a reported malignancy rate of 38-70%. Within an anti-TIF1-γ positive cohort, breast cancer was the most common malignancy (33%), followed by ovarian cancer (19%) and lymphoma (14%) (22). Among 72 IIM patients with malignancies in a cohort study from Northern China, 38 tested positive for anti-TIF1-γ, 3 for anti-NXP2, 4 for anti-SAE1, 10 for anti-ARS, 1 each for anti-MDA5, anti-HMGCR, and anti-SRP (23). The most common tumor types in MSA positive patients were lung, gynecological, breast, gastrointestinal and nasopharynx cancers sequentially. Similarly, the most common malignancy-associated antibody in our cohort study was not surprisingly anti-TIF1-γ, followed by anti-Mi-2. Interestingly, NPC was the most common malignant tumor, followed by thyroid and other cancers. NPC has also been found to be the most frequent malignancy associated with DM in Malaysia, Singapore and other Asian countries (24–26). Unlike anti-TIF1-γ, the association between anti-Mi-2 antibody and cancer in IIM patients remains unclear. Several studies have shown that anti-Mi-2 is either not associated with the development of malignancies in patients with IIMs or is a negative risk factor (23, 27). However, some studies have found that patients with anti-Mi-2 have an increased risk of cancer (28, 29). Although anti-Mi-2 was the second most common antibody detected in cancer patients after anti-TIF1-γ in this study, further analysis revealed that two of the three cancer patients with positive anti-Mi-2 were also positive for anti-TIF1-γ. Therefore, the correlation between Mi-2 and cancers warrants further study with a larger sample size and to exclude the interference of multiple positive antibodies.

ILD presents in 20-80% IIM patients. The incidence varies according to the studied population and methods used to identify ILD (10, 30). In our cohort, 71.1% IIM patients presented with ILD. Patients with ILD had a higher incidence of fever, arthritis, mechanic’s hands and myalgia which were consistent with characteristics of the anti-MDA5 and anti-ARS group. However, the myalgia here may not necessarily be a manifestation of severe muscle damage but may be related to a high inflammatory state or pain from other tissue around the muscles such as skin and joints. MSAs analysis in IIM patients with/without ILD revealed significant different positive rate of anti-ARS, anti-HMGCR, anti-MDA5, anti-NXP2, anti-Ro52, and anti-TIF1-γ. Further logistic regression analysis of MSA showed that only anti-MDA5 and anti-ARS antibodies were risk factors for ILD. Anti-HMGCR, anti-TIF1-γ and anti-NXP2 were negatively correlated with ILD but lacked statistical significance. The positive rate of anti-Ro52 was indeed higher in the ILD group than non-ILD group, but it always co-exists with other MSAs, especially anti-MDA5 and anti-ARS. It is not a risk factor for ILD independently in our study. The combination of anti-Ro52 with anti-MDA5 or anti-ARS has been reported to be associated with more severe ILD and poorer outcomes, and/or higher mortality (31–33).

The differences in clinical characteristics between MSA antibody subgroups provide a theoretical basis for more targeted and personalized treatment. As we have found, compared with group III, group I and group II, particularly group I, often exhibit more pronounced inflammation, even with the development of a cytokine storm associated with macrophage activation syndrome (MAS). In these patients, potent anti-inflammatory drugs such as JAK inhibitor and IL-6 inhibitor could be effective in disease control and glucocorticoids reduction. There have been many reports that tofacitinib is used to treat RP-ILD and calcineurin inhibitors targeting activated T cells are classic immunosuppressants used to treat IIM-related ILD (34–37).

Of course, this study has certain limitations. A major limitation is the consolidation of several clinically distinct MSAs (e.g., anti-SRP, anti-TIF1-γ) into a single heterogeneous “others” group, which may mask some less frequent but still clinically meaningful differences. However, as previously mentioned, we partially compensated for this limitation by performing a sub-analysis within Group III.

In conclusion, MSA stratification is essential for diagnosing, treating, and predicting outcomes in IIM. Anti-MDA5 and anti-ARS antibodies are independent risk factors for ILD, each with distinct clinical implications. The high malignancy risk in anti-TIF1-γ and anti-Mi-2 positive patients and high percentage of cardiac involvement and hyperlipidemia in group III patients highlight the need for targeted screening and monitoring. Personalized therapeutic strategies, including immunosuppressants and biologics, are crucial for improving outcomes, particularly in patients with severe organ involvement. Future large-scale, multicenter studies are needed to validate these findings and explore novel therapeutic targets.

## Data Availability

The original contributions presented in the study are included in the article/**Supplementary Material**. Further inquiries can be directed to the corresponding author.

## Supplementary material

Supplementary Figure 1 The Multi-Positive Antibody Profiles.

Supplementary Table 1 Basic clinical features among IIM patients.

Supplementary Table 2 Clinical Characteristics of IIM Patients with Multi-MSAs.

Supplementary Table 3 Malignancy Screening and Outcomes in IIM Patients.

Supplementary Table 4 Baseline Pulmonary Function Test in IIM-ILD Patients.

Supplementary Table 5 Treatment regimens for patients with IIM.
