# Correction: Association of clinical features and myositis-specific antibodies in idiopathic inflammatory myopathy: a retrospective study from southern China

**DOI:** 10.3389/fimmu.2025.1743471

**Published:** 2025-12-15

**Authors:** Can Li, Yushi Zheng, Yu Zhang, Yujin Ye, Hui Zhang, Niansheng Yang, Shuang Wang

**Affiliations:** 1 Department of Rheumatology, First Affiliated Hospital of Sun Yat-sen University, Guangzhou, China; 2 Department of Medicine, Traditional Chinese Medicine Hospital of Laifeng County, Enshi, Hubei, China

**Keywords:** idiopathic inflammatory myopathy, myositis-associated autoantibody, myositis-specific autoantibody, interstitial lung disease, malignancy, cardiac involvement, hyperlipidemia

There was a mistake in **Figure 1C** as published. The original, incorrect text was “MSA(+)&MAA(-)”. The correct text should be “MSA(+)&MAA(±)”. The corrected **Figure 1** appears below.

**Figure 1:**
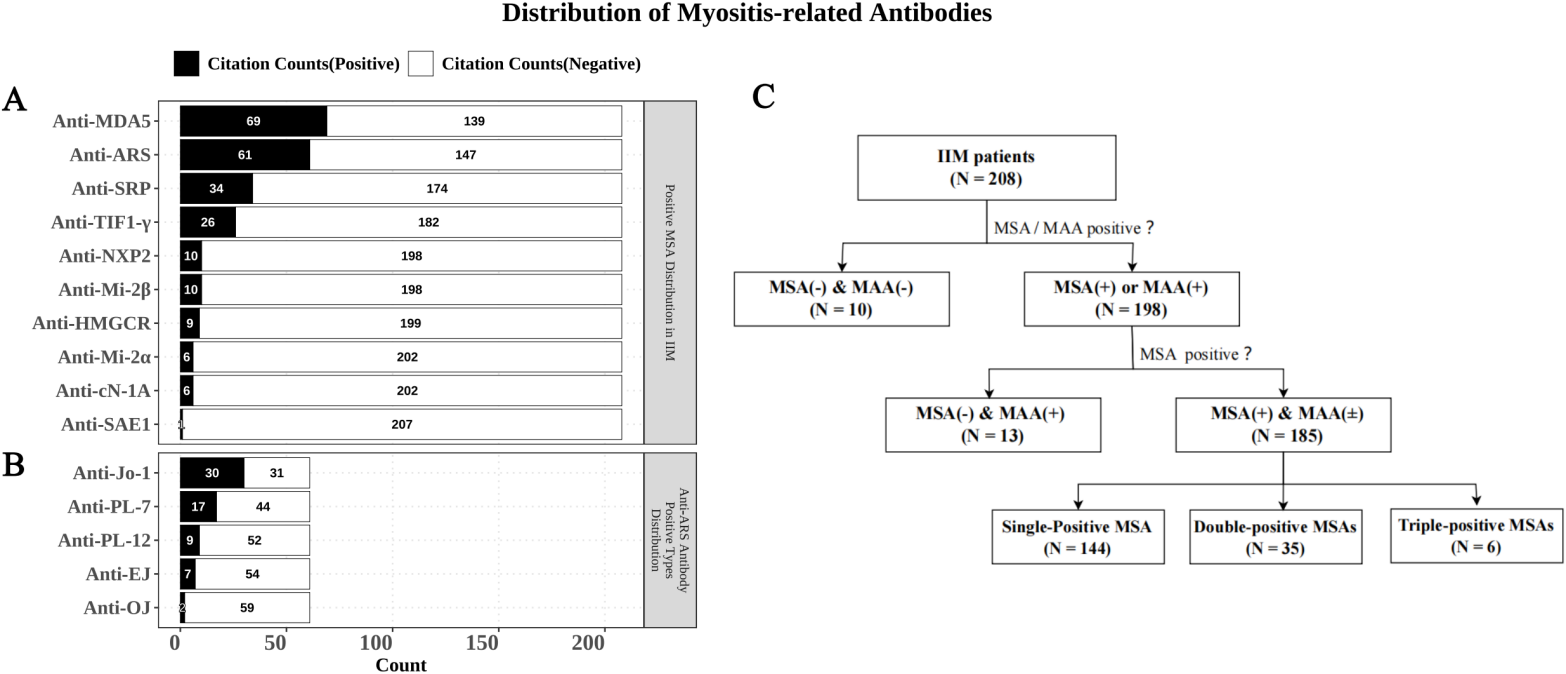
The distribution of myositis-related antibodies in IIM patients. **(A)** The distribution of MSAs in 208 IIM patients. **(B)** Distribution of various anti-ARS antibodies in anti-ARS positive patients. **(C)** Profiles of MSA and MAA positivity in IIM patients.

The original version of this article has been updated.

