# Supplementary figures and images for: Association of clinical features and myositis-specific antibodies in idiopathic inflammatory myopathy: a retrospective study from southern China
Source: Front Immunol. 2025 Nov 6;16:1674437. doi: 10.3389/fimmu.2025.1674437 (PMC12631342; doi:10.3389/fimmu.2025.1674437)

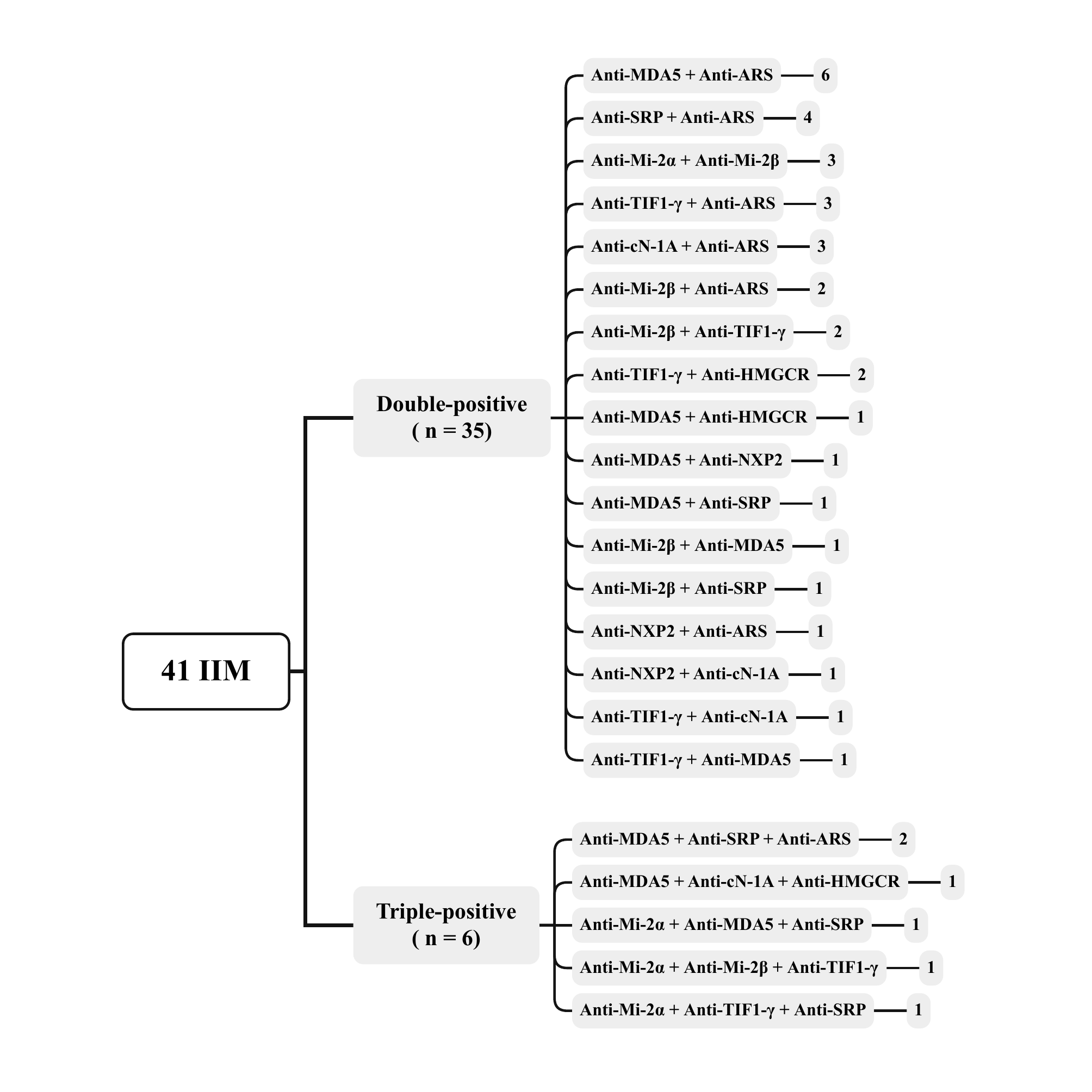

Supplement: Supplementary Figure 1 — The Multi-Positive Antibody Profiles. [file Image1.tif]
