# Supplementary material for: Association of clinical features and myositis-specific antibodies in idiopathic inflammatory myopathy: a retrospective study from southern China
Source: Front Immunol. 2025 Nov 6;16:1674437. doi: 10.3389/fimmu.2025.1674437 (PMC12631342; doi:10.3389/fimmu.2025.1674437)
Supplement: Supplementary Table 1 — Basic clinical features among IIM patients. [file Table1.docx]

**Supplementary material**

Table S1 Basic clinical features among IIM patients

| **Category** | **IIM Cohort (N = 208)** |
| --- | --- |
| Sex (Female) | 145 (69.7%) |
| Disease Duration (months) | 6.00 (3.00，24.0) |
| Age (years) | 50.0 (41.0，57.2) |

For skewed continuous variables, the median (25th, 75th percentiles) is used, and categorical variables are presented as counts (percentages).
