# Supplementary material for: Association of clinical features and myositis-specific antibodies in idiopathic inflammatory myopathy: a retrospective study from southern China
Source: Front Immunol. 2025 Nov 6;16:1674437. doi: 10.3389/fimmu.2025.1674437 (PMC12631342; doi:10.3389/fimmu.2025.1674437)
Supplement: Supplementary Table 2 — Clinical Characteristics of IIM Patients with Multi-MSAs. [file Table2.docx]

Table S2 Clinical Characteristics of IIM Patients with Multi-MSAs

| **Clinical Characteristics of IIM Patients with Multi-MSAs (N = 41)** | |
| --- | --- |
| **Demographics** |  |
| **Survival Status** |  |
| - Alive | 37 (90.2%) |
| - Dead | 3 (7.3%) |
| - Lost to follow-up | 1 (2.4%) |
| **Gender, n (%)** |  |
| - Female | 27 (65.9%) |
| - Male | 14 (34.1%) |
| **Age (years)** | 48.0 (17-73) |
| **Disease Duration (months)** | 6.0 (1-223) |
| **Clinical Manifestations** |  |
| - Muscle Weakness | 22 (53.7%) |
| - Muscle Pain | 17 (41.5%) |
| - Dysphagia for Water | 5 (12.2%) |
| - Swallowing Difficulty | 7 (17.1%) |
| - Dyspnea | 1 (2.4%) |
| - Heliotrope Rash | 18 (43.9%) |
| - Gottron's Sign/Papules | 11 (26.8%) |
| - Shawl Sign | 4 (9.8%) |
| - Mechanic's Hands | 4 (9.8%) |
| - Raynaud's Phenomenon | 1 (2.4%) |
| - Skin Ulcers | 1 (2.4%) |
| - Arthritis | 17 (41.5%) |
| - Alopecia | 4 (9.8%) |
| - Fever | 3 (7.3%) |
