# Supplementary material for: Association of clinical features and myositis-specific antibodies in idiopathic inflammatory myopathy: a retrospective study from southern China
Source: Front Immunol. 2025 Nov 6;16:1674437. doi: 10.3389/fimmu.2025.1674437 (PMC12631342; doi:10.3389/fimmu.2025.1674437)
Supplement: Supplementary Table 3 — Malignancy Screening and Outcomes in IIM Patients. [file Table3.docx]

Table S3 Malignancy Screening and Outcomes in IIM Patients

| **Malignancy** | **Ascertainment window^#^** | **Screening modality** | **Survival status** |
| --- | --- | --- | --- |
| Nasopharyngeal carcinoma 1 | -3 years | Nasopharyngoscopy | Alive |
| Nasopharyngeal carcinoma 2 | -1 year | Nasopharyngoscopy | Alive |
| Nasopharyngeal carcinoma 3 | -15 years | Nasopharyngoscopy | Dead |
| Nasopharyngeal carcinoma 4 | 0 | Whole-body PET-CT | Alive |
| Nasopharyngeal carcinoma 5 | +4 months | Nasopharyngoscopy | Alive |
| Nasopharyngeal carcinoma 6 | 0 | Whole-body PET-CT | Alive |
| Nasopharyngeal carcinoma 7 | 0 | Whole-body PET-CT | Alive |
| Nasopharyngeal carcinoma 8 | 0 | Whole-body PET-CT | Alive |
| Nasopharyngeal carcinoma 9 | +2 years | Whole-body PET-CT | Alive |
| Thyroid carcinoma 1 | 0 | Thyroid ultrasound | Alive |
| Thyroid carcinoma 2 | 0 | Thyroid ultrasound | Alive |
| Thyroid carcinoma 3 | 0 | Thyroid ultrasound | Alive |
| Thyroid carcinoma 4 | +7 years | Thyroid ultrasound | Alive |
| Breast Cancer | 0 | Breast ultrasound | Alive |
| Colon Cancer | 0 | Abdominal CT | Alive |
| Hypopharyngeal carcinoma | 0 | Whole-body PET-CT | Dead |
| Endometrial carcinoma | 0 | Tumour markers (CA125, CA19-9) | Dead |

^#^The ascertainment window is the time from the diagnosis of the malignancy to the onset of myositis. "+" represents the cancer was diagnosed after the onset of myositis, "–" represents the cancer was diagnosed before onset of myositis. " 0" represents the cancer was diagnosed at the same time as the myositis onset.
