# Supplementary material for: Association of clinical features and myositis-specific antibodies in idiopathic inflammatory myopathy: a retrospective study from southern China
Source: Front Immunol. 2025 Nov 6;16:1674437. doi: 10.3389/fimmu.2025.1674437 (PMC12631342; doi:10.3389/fimmu.2025.1674437)
Supplement: Supplementary Table 4 — Baseline Pulmonary Function Test in IIM-ILD Patients. [file Table4.docx]

Table S4 Baseline Pulmonary Function Test in IIM-ILD Patients.

|  | **Group I-ILD**  **(N = 45)** | **Group II-ILD**  **(N = 35)** | **Group III-ILD**  **(N = 24)** | **p-value** |
| --- | --- | --- | --- | --- |
| FVC% pred (Mean ± SD) | 74.4 (13.0) | 68.7 (18.5) | 76.7 (17.1) | 0.297 |
| DLCO% [25th, 75th] | 52.0 [49.2, 71.0] | 58.5 [52.0, 66.5] | 79.0 [74.5, 86.5] | 0.013 |
