# Supplementary material for: Association of clinical features and myositis-specific antibodies in idiopathic inflammatory myopathy: a retrospective study from southern China
Source: Front Immunol. 2025 Nov 6;16:1674437. doi: 10.3389/fimmu.2025.1674437 (PMC12631342; doi:10.3389/fimmu.2025.1674437)
Supplement: Supplementary Table 5 — Treatment regimens for patients with IIM. [file Table5.docx]

Table S5 Treatment Regimens for Patients with IIM

| *Medication* | *Group I* | | *Group II* | | *Group III* | |
| --- | --- | --- | --- | --- | --- | --- |
|  | *ILD*  *(N = 45)* | *non-ILD*  *(N = 5)* | *ILD*  *(N = 35)* | *non-ILD*  *(N = 3)* | *ILD*  *(N = 24)* | *non-ILD*  *(N = 37)* |
| Corticosteroids | 45 (100.0%) | 5 (100.0%) | 35 (100.0%) | 3 (100.0%) | 24 (100.0%) | 37 (100.0%) |
| Cyclophosphamide | 9 (20.0%) | 1 (20.0%) | 7 (20.0%) | 0 (0.0%) | 6 (25.0%) | 10 (27.0%) |
| Mycophenolate Mofetil | 28 (62.2%) | 3 (60.0%) | 22 (62.9%) | 3 (100.0%) | 9 (37.5%) | 11 (29.7%) |
| Methotrexate | 1 (2.2%) | 0 (0.0%) | 0 (0.0%) | 0 (0.0%) | 4 (16.7%) | 7 (18.9%) |
| Cyclosporine | 1 (2.2%) | 1 (20.0%) | 2 (5.7%) | 0 (0.0%) | 2 (8.3%) | 1 (2.7%) |
| Tacrolimus | 14 (31.1%) | 0 (0.0%) | 2 (5.7%) | 0 (0.0%) | 0 (0.0%) | 2 (5.4%) |
| Tofacitinib | 2 (4.4%) | 0 (0.0%) | 1 (2.9%) | 0 (0.0%) | 0 (0.0%) | 1 (2.7%) |
| Baricitinib | 1 (2.2%) | 0 (0.0%) | 0 (0.0%) | 0 (0.0%) | 0 (0.0%) | 0 (0.0%) |
| Hydroxychloroquine | 18 (40.0%) | 1 (20.0%) | 3 (8.6%) | 1 (33.3%) | 7 (29.2%) | 10 (27.0%) |
| Thalidomide | 1 (2.2%) | 0 (0.0%) | 0 (0.0%) | 0 (0.0%) | 2 (8.3%) | 4 (10.8%) |
| Rituximab | 1 (2.2%) | 0 (0.0%) | 0 (0.0%) | 0 (0.0%) | 0 (0.0%) | 0 (0.0%) |
| Plasma Exchange | 4 (8.9%) | 0 (0.0%) | 0 (0.0%) | 0 (0.0%) | 1 (4.2%) | 1 (2.7%) |
| Immunoglobulin | 12 (26.7%) | 0 (0.0%) | 4 (11.4%) | 0 (0.0%) | 4 (16.7%) | 7 (18.9%) |
